# Review of Perioperative Care Pathway for Children With Renal Tumors

**DOI:** 10.7759/cureus.24928

**Published:** 2022-05-11

**Authors:** Sajid Ali, Tariq Latif, Muhammad Ali Sheikh, Muhammad Bilal Shafiq, Dur-e-Zarnab Zahra, Muhammad Abu Bakar

**Affiliations:** 1 Pediatric Surgery, Shaukat Khanum Memorial Cancer Hospital and Research Centre, Lahore, PAK; 2 Surgical Oncology, Shaukat Khanum Memorial Cancer Hospital and Research Centre, Lahore, PAK; 3 Pediatric Surgery, Shaikh Zayed Postgraduate Medical Institute, Lahore, PAK; 4 Surgery, Shaukat Khanum Memorial Cancer Hospital and Research Centre, Lahore, PAK; 5 Biostatistics and Epidemiology, Shaukat Khanum Memorial Cancer Hospital and Research Center, Lahore, PAK

**Keywords:** outcome, eras, nephrectomy, renal tumors, children

## Abstract

Introduction

Wilms tumor is the most common (90%) renal tumor in children. With the recent advances survival rate approaches 90%. This study was designed to identify factors associated with early recovery and hospital discharge, a step forward in the future development of early recovery after surgery (ERAS) protocol in children.

Methods

A retrospective cohort study was conducted from May 2020 to February 2022 among children up to the age of 18-years with a diagnosis of primary malignant renal tumors who underwent radical nephrectomy. Baseline parameters (preoperative), intraoperative, and postoperative components were analyzed. A univariable and multivariable logistic regression model was used to identify the factors leading to early discharge. The data were entered into SPSS version 20 (IBM Inc., Armonk, USA), and a p-value ≤0.05 was statistically significant.

Results

A total of 44 patients with malignant renal tumors were managed with a mean age at diagnosis of 4.06±3.00 years. Twenty-six (59.1%) were male and 18 (40.9%) were female. All the patients received neoadjuvant chemotherapy. Wilms tumor was the most common pathology found in 41 (93.1%) patients; 19 (43.0 %) had stage I, 21 (47.7%) stage II, and four (9.1%) stage III disease. Thirty-four (77.3%) patients had a nasogastric tube placed postoperatively. Median blood loss (BL) was 40 ml (range: 5-250), and the median operative time was two hours (range: 1-4). The median postoperative day to mobilization was one day (range: 1-3), to clear liquids was two days (range: 1-3), and advanced to regular diet was three days (range: 2-5). The median postoperative day of discharge (from surgery to discharge) was four days (range: 2-7), with 31 (70.5%) patients discharged early.

Conclusion

Our findings indicate that early removal of tube, mobilization, and re-feedings were significantly associated with the early hospital discharge, while the other analyzed factors were not statistically significant. Furthermore, our findings are important in the future development and implementation of ERAS protocol in pediatric oncological resections.

## Introduction

Childhood renal tumors account for 7% of all cancers. Almost 6% of all childhood malignancies are Wilms tumor (nephroblastoma) and one of the most common (90%) renal tumors in children. In recent times advances (in surgical technique and chemotherapy) resulted in great improvement in prognosis and overall survival [1,2]. Radical nephrectomy with or without neoadjuvant treatment remains the standard of care. Then typically, adjuvant therapy follows, with chemotherapy and radiation as dictated by surgical staging and histopathology. However, early recovery and timely initiation of adjuvant therapy is a specific requirement after radical nephrectomy, specifically in children, and that should be initiated as early as possible, within two weeks of surgery [3]. Therefore, factors that can enable early recovery and short hospital stay can maximize protocol adherence concerning the timing of adjuvant therapy initiation.

Enhanced recovery after surgery (ERAS) is a multidisciplinary approach, a pathway designed to improve patient care, expedite surgical recovery, and reduce postoperative complications [4]. ERAS protocols have been introduced initially in adults to decrease surgical stress, facilitate postoperative recovery and improve outcomes. Several studies have shown the implementation of ERAS protocols in adults with decreased complications rate, length of hospital stay, and costs in adults undergoing a variety of different surgeries like radical cystectomy, colorectal surgery, gastrectomy, pelvic surgery, and pancreaticoduodenectomy [5-10]. However, the role of ERAS protocols for children is still evolving, and few publications have shown that it's safe, feasible, facilitates shorter postoperative stay, and is associated with high parental satisfaction [11,12].

A systematic review by Vukovic and Dinic (2018) reported that there are still scarce guidelines for ERAS protocols in major urologic surgery, which is why further studies should assess the importance of preoperative optimization, perioperative nutritional management, and implementation of advanced anesthesia and analgesia [13]. A recently published study by Saltzman et al. on the postoperative care pathway for children with renal tumors documented the significance of various factors like early mobilization and pain control associated with early recovery and shorter hospital stay with better results [14].

Therefore, in the developing world with limited published data, a step forward toward the future development of ERAS protocol in children undergoing surgery for various oncological reasons, the current study is designed to identify factors associated with early recovery and short hospital stay, which helps in timely initiation of adjuvant therapy and preserve the hospital resources. Furthermore, it will also be helpful to modify the traditional practices of perioperative care to provide better outcomes in major pediatric oncological resections.

## Materials and methods

This is a retrospective cohort study carried out at the Department of Surgical Oncology at Shaukat Khanum Memorial Cancer Hospital and Research Centre, Lahore, Pakistan, from May 2020 to February 2022. Institutional Review Board (IRB) approval was obtained (IRB No: EX-04-03-22-02). All the pediatric patients up to the age of 18 years with malignant renal tumors who underwent radical nephrectomy were included.

Cases were identified from electronic records of the hospital information system (HIS). All findings were recorded in pre-designed forms. Variables that were analyzed included demographic details (age, sex, tumor laterality), tumor volume, preoperative chemotherapy and bowel preparation, antibiotic prophylaxis, perioperative findings and lymph node sampling, stage, operative time, estimated blood loss and transfusion, a postoperative nasogastric tube (NGT) and abdominal drain (AD) used, median postoperative day (POD) for mobilization on a bed, NGT, Foleys catheter and drain removal, the time to start of clear liquids and regular diet, and hospital discharge. The median time from surgery to discharge (median postoperative day) was used as the benchmark and the patients discharged at and before the median postoperative day were recorded as early discharge, and those who stayed after median postoperative day were recorded as late discharge. Thirty days of postoperative complications, emergency room visits, and readmissions were also recorded.

The IBM SPSS version 20 (IBM Inc., Armonk, USA) was utilized for statistical analysis. Numerical variables were reported as medians or mean (range: minimum-maximum or standard deviation), and categorical data were presented as frequencies and percentages. The Mann-Whitney U test, t-test, Fisher's exact test, and Chi-square test were applied to analyze the differences between groups (early versus late discharge) according to the type of data. A univariable and multivariable logistic regression model was applied to identify the factors leading to early discharge. The differences between groups were considered to be statistically significant when the p-value was ≤0.05.

## Results

A total of 44 patients were reviewed who underwent radical nephrectomy for renal tumors. The mean age at diagnosis was 4.06±3.00 years; 26 (59.1%) were male, and 18 (40.9%) were female. Wilms tumor was the most common pathology (n=41, 93.1%), two with clear cell sarcoma and one with Ewing sarcoma of the kidney. Stage distribution, as per the International Society of Paediatric Oncology (SIOP) staging system, was 19 (43.0%) patients with stage I, 21 (47.7%) - stage II, and four (9.1%) - stage III. All the patients received neoadjuvant chemotherapy. All the patients received antibiotic prophylaxis, and eight (18.2%) had bowel preparation before surgery. Combined central venous access was placed in six (13.6%) patients, and 34 (77.3%) had nasogastric tube placement. Combined multimodal analgesia was used in the 19 (43.2%) with epidural block and nurse controlled analgesia (NCA), eight (18.2%) had an epidural block, and 17 (38.6%) had NCA along with other non-opioids analgesics. The median tumor volume was 7.90 cm (range: 1-15). The median lymph nodes (LN) yield was two (range: 1-10). Median blood loss (BL) was 40 ml (range 5-250). The median operative time was two hours (range: 1-4). The median postoperative day of discharge (from surgery to discharge) was four days (range: 2-7). Data were further stratified concerning the median postoperative day of discharge (POD 4) into early and late discharge, and 31 (70.5%) patients were discharged at or before POD 4 (early discharge). Demographic and baseline clinical information is summarized in Table 1. Only two patients had postoperative complications during the same admission - one had loose stools, and the other had a fever. There was no history of readmissions or emergency room visits during the 30-day follow-up.

**Table 1:** Demographics and clinical information of children with renal tumors ROD - postoperative day; NGT - nasogastric tube

	Total (n=44)	Early discharge (n=31; 70.5%)	Late discharge (n=13; 29.5%)	p-value
Age (years)				0.24
Mean ± SD	4.06 ± 3.00	5.31 ± 3.54	3.93 ± 3.41	
Gender				0.26
Male	26 (59.1%)	20 (64.5%)	6 (46.2%)	
Female	18 (40.9%)	11 (35.3%)	7 (53.8%)	
Tumor laterality				0.32
Right side	22 (50.0%)	14 (45.2%)	8 (61.5%)	
Left side	22 (50.0%)	17 (54.8%)	5 (38.5%)	
Histology				0.65
Wilms tumor	41 (93.1%)	28 (89.3%)	12 (80.0%)	
Clear cell sarcoma	2 (4.5%)	1 (3.6%)	1 (6.7%)	
Ewing sarcoma	1 (2.3%)	1 (3.6%)	-	
Stage at diagnosis				0.03
Stage I	19 (43.0%)	16 (51.1%)	3 (23.1%)	
Stage II	21 (47.7%)	14 (46.7%)	7 (53.8%)	
Stage III	4 (9.10%)	1 (3.30%)	3 (23.1%)	
Preoperative management				
Bowel preparation				0.4
Yes	8 (18.2%)	7 (22.6%)	1 (7.7%)	
No	36 (81.8%)	24 (77.4%)	12 (92.3%)	
Intraoperative management				
Combined central venous access placement				0.65
Yes	6 (13.6%)	5 (16.1%)	1 (7.7%)	
No	38 (86.4%)	26 (83.9%)	12 (92.3%)	
Type of analgesia				0.82
Epidural block	8 (18.2%)	5 (42.9%)	3 (40.0%)	
Nurse controlled analgesia	17 (38.6%)	12 (38.7%)	5 (38.5%)	
Combined	19 (43.2%)	14 (45.0%)	5 (38.0%)	
NGT placement				0.24
Yes	34 (77.3%)	22 (71.0%)	12 (92.3%)	
No	10 (22.7%)	9 (29.0%)	1 (7.7%)	
Lymph node sampling				0.46
Yes	33 (75.0%)	22 (71.0%)	11 (84.6%)	
No	11 (25.0%)	9 (29.0%)	2 (15.4%)	
Blood transfusion				1
Yes	2 (4.5%)	2 (6.5%)	-	
No	42 (95.5%)	29 (93.5 %)	13 (100 %)	
Median mlood loss, ml (range)	40 (5-250)	30 (5-200)	50 (20-250)	0.12
Median operative time, hours (range)	2 (1-4)	1.5 (1-3)	2 (1-4)	0.06
Median lymph node yield (range)	2 (1-10)	1 (1-8)	2 (1-10)	0.43
Postoperative management				
Median POD for NGT removal (range)	1.00 (1-3)	1 (1-2)	2 (1-3)	0.08
Median POD for liquids (range)	2.00 (1-3)	1 (1-3)	2 (1-3)	0.05
Median POD for regular diet (range)	3.00 (2-5)	2 (2-3)	3 (2-5)	0.001
Median POD for drain removal (range)	2.00 (1-3)	1 (1-3)	2 (2-3)	0.05
Median POD for catheter removal (range)	2.00 (1-5)	1 (1-3)	2 (1-5)	0.009
Median POD of mobilization (range)	1.00 (1-3)	1 (1-3)	2 (1-3)	0.02
Median POD of discharge (range)	4.00 (2-7)	4 (2-4)	5 (5-7)	0.001

Furthermore, median operative time, postoperative day (POD) of mobilization on a bed, removal of Foley's catheter, advanced to oral liquids, and regular diet were statistically significant with early discharge based on univariable logistic regression with a p-value of ≤0.05. Age of patients and postoperative day to regular diet (p-value ≤0.05) was positively associated with early discharge on multivariable logistic regression, while the remaining analyzed factors were not statistically significant. Univariable and multivariable logistic regression analyses are summarized in Table 2. 

**Table 2:** Adjusted logistic regression analysis of factors associated with early discharge ROD - postoperative day; NGT - nasogastric tube

Variables	Univariable logistic regression model			Multivariable logistic regression model		
	Odds ratio	Confidence interval	p-value	Odds ratio	Confidence interval	p-value
Age (years)	0.89	0.75-1.07	0.24	0.63	0.40-0.99	0.04
Tumor volume	0.81	0.65-1.01	0.06	0.94	0.61-1.47	0.82
Operative time	0.38	0.14-1.03	0.05	0.48	0.08-2.78	0.41
Lymph node yield	0.88	0.66-1.18	0.42	0.55	0.26-1.18	0.13
Blood loss	0.99	0.98-1.01	0.39	1.00	0.98-1.02	0.91
POD mobilization	0.28	0.09-0.89	0.03	0.65	0.08-2.78	0.49
POD removal NGT	0.35	0.11-1.11	0.07	0.73	0.05-9.10	0.80
POD catheter removal	0.29	0.11-0.80	0.01	0.31	0.02-4.37	0.38
POD regular diet	0.06	0.01-0.49	0.01	0.05	0.02-4.37	0.03

## Discussion

With the advancement of the multidisciplinary collaborative group approach for the management of children with Wilms tumors, the five-year survival rate approaches up to 90%; recent investigational priorities are focused to achieve the optimal results with safety and reduction in treatment-related morbidity [15]. Surgery follows the risk group stratification, and adjuvant chemotherapy is to be given once peristalsis is established [16]. Thus, factors that can assist in early recovery and discharge from the surgical facility can maximize protocol adherence with timely initiation of adjuvant therapy.

This study reviewed the various factors related to rapid recovery and early discharge in children who underwent nephrectomy. Specifically, tumor histology and volume, preoperative bowel preparation, lymph node sampling and yield, operative time, blood loss, preoperative chemotherapy, and antibiotic prophylaxis were not significant with early discharge. Moreover, our findings are comparable with other similar studies, with limited use and early removal of various tubes, including nasogastric tubes (NGT), abdominal drains, and Foley's catheters facilitating the early mobilization, pain control and suggesting the abandonment of the routine use of postoperative NGT and abdominal drains [17-19].

Rapid recovery of gastrointestinal function has been associated with early mobilization, enteral feeding, and, ultimately, hospital discharge [20,21], and these factors were positively seen in our results with early hospital discharge and less complication rate. Thus, it seems reasonable that while developing early recovery after surgery (ERAS) protocol for children, these factors should be considered specifically.

In the proposed adult colorectal literature, bowel preparation is associated with a 50% reduction in postoperative complications and hospital readmission [22]. However, these findings were not found in the urological literature [23]; similarly, bowel preparation does not positively correlate with early discharge in our results.

In our study, combined multimodal analgesia was used in the majority of patients with epidural block and nurse controlled analgesia along with other non-opioid analgesics, but the results were not significant due to the small sample size; however, there is clear evidence suggesting that the use of multimodal analgesia and epidural infusion in children reduces the parenteral opioid requirements, possibly contributes to the early return of gut motility and decrease the duration of hospital stay [24,25].

Different studies have shown the importance of perioperative temperature in ERAS, especially in children [26]. In our study, all patients underwent intraoperative warming with the help of warm beds and warmers while the postoperative temperature was controlled with paracetamol, so the significance of perioperative warming cannot be compared in our cohort. Similarly, some authors have defined the role of antiemetics in enhanced recovery after abdominal surgery in children [27]. However, all patients in our study were prescribed dimenhydrinate for 24 hours; the effect of this medication could not be measured in our cohort.

In a literature review, the use of ERAS in the pediatric population is still growing, and most published studies have investigated few elements of the ERAS in a specific population group, so results of the application of an entire protocol are absent [28]. A single study in pediatric colorectal surgery demonstrated that ERAS implementation is safe and feasible and may lead to improved outcomes with a decreased hospital stay, narcotic use, time on a regular diet, and intraoperative fluid volumes [29].

Although this study is an experience from a single center with limited data, it emphasizes the implementation of ERAS protocol in this specific age group without increasing the risk of complications. Further prospective collaborative studies will be valuable to aid more knowledge and validate the concept for future directions and betterment of patient management and outcome.

## Conclusions

The data suggest that early tubes removal, mobilization, and re-feedings were significantly associated with the early hospital discharge, while the other analyzed factors were not associated with early discharge. Furthermore, our findings are important in the future development and implementation of ERAS protocol in pediatric oncological resections. It is safer and more feasible, will reduce the parent's anxiety and hospital costs, and accelerate postoperative recovery without increasing the risk of complications.
